# An Electrochemical Impedimetric Aptasensing Platform for Sensitive and Selective Detection of Small Molecules Such as Chloramphenicol

**DOI:** 10.3390/s140712059

**Published:** 2014-07-07

**Authors:** Sanaz Pilehvar, Tarryn Dierckx, Ronny Blust, Tom Breugelmans, Karolien De Wael

**Affiliations:** 1 AXES Research Group, Department of Chemistry, University of Antwerp, Groenenborgerlaan 171, 2020 Antwerp, Belgium; E-Mail: sanaz.pilehvar@uantwerpen.be; 2 ART Research Group, Faculty of Applied Engineering, University of Antwerp, Salesianenlaan 90, 2000 Antwerp, Belgium; E-Mails: tarryn.dierckx@gmail.com (T.D.); tom.breugelmans@uantwerpen.be (T.B.); 3 Sphere Research Group, Department of Biology, University of Antwerp, Groenenborgerlaan 171, 2020 Antwerp, Belgium; E-Mail: ronny.blust@uantwerpen.be

**Keywords:** aptasensor, electrochemical impedance spectroscopy, chloramphenicol, specific recognition

## Abstract

We report on the aptadetection of chloramphenicol (CAP) using electrochemical impedance spectroscopy. The detection principle is based on the changes of the interfacial properties of the electrode after the interaction of the ssDNA aptamers with the target molecules. The electrode surface is partially blocked due to the formation of the aptamer-CAP complex, resulting in an increase of the interfacial electron-transfer resistance of the redox probe detected by electrochemical impedance spectroscopy or cyclic voltammetry. We observed that the ratio of polarization resistance had a linear relationship with the concentrations of CAP in the range of 1.76–127 nM, and a detection limit of 1.76 nM was obtained. The covalent binding of CAP-aptamer on the electrode surface combined with the unique properties of aptamers and impedimetric transduction leads to the development of a stable and sensitive electrochemical aptasensor for CAP.

## 1. Introduction

Chloramphenicol (CAP), an effective and broad-spectrum phenicol antibiotic, has been found to have potential lethal side effects on humans, such as leukemia, aplastic anemia and grey baby syndrome [1]. Therefore, to ensure the food safety products, the European Commission has set a minimum required performance limit (MRPL) value for CAP at a level of 0.3 × 10^−6^ g·kg^−1^ [2]. Consequently, a rapid, accurate, simple and inexpensive detection method for sensing this low amount of CAP is required. Currently used detection methods for CAP and its derivatives are based on gas chromatography, high performance liquid chromatography and mass spectrometry [3–5]. These methods are very sensitive for the MRPL value, but they are time consuming, expensive and they require highly trained personnel. For these reasons the need arises for the development of a new and fast analytical method. Electrochemical biosensors are widely used due to their low cost, high time efficiency and simple operation. In addition to these advantages, these sensors show a higher sensitivity than the classical methods and they have great potential for the construction of portable devices [6,7].

Electrochemical biosensors consist of a biological recognition element to target the desired analyte and a transduction element to convert the detection into a measurable signal [6]. Since the invention of SELEX methodology, aptamers are becoming increasingly important as a new class of recognition element within the field of biosensors [8,9]. Aptamers are single-stranded synthetic oligonucleic acids that bind with high affinity and specificity to target molecules. Moreover, they have superior properties such as high specificity, stability, accuracy and reproducible production [8–10].

Pilehvar *et al.* developed an amperometric CAP sensor, based on CAP-specific aptamers, that approaches the MRPL value and that was able to detect CAP in the presence of its analogues [11,12]. Later on, Yan *et al.* developed an amperometric aptasensor for the detection of CAP based on target-induced strand release (TISR). The detection limit for CAP was found to be 0.29 nM [13]. However, the electrochemical impedance spectroscopy (EIS) is believed to be more favorable than other electrochemical detection techniques since significantly large differences in low target concentration range are usually obtained due to the inverse relation of the impedance with the current (ΔR (Z) = ΔV/ΔI) [14,15]. One of the biggest advantages of EIS lies in the fact that the electrochemical system is only perturbed within a region of 10 mV or smaller. The signal is small enough to confine the system as a pseudo-linear segment and the measurements of non-linear response (capacitance current) are less pronounced. In addition, due to this small perturbation the electrochemical system can be perceived as stationary and measurements with high precision are possible. Therefore, the binding between aptamer and target is not influenced and the response can be described by simple linear current-potential characteristics. In comparison with amperometry, detailed knowledge of the current-potential curve for a wide potential range is not necessary for EIS and important simplifications can be made for the kinetic and diffusion phenomena [8,16]. For these reasons, we have now focused on the EIS as a technique for CAP aptasensing.

We report a direct and label-free impedimetric aptasensing device that not only can sensitively detect CAP, but that moreover allows the unravelling of the different subprocesses occurring during the binding of CAP and the aptamers by using an electrical equivalent circuit (EEC). The fitting of the circuit is statistically analyzed and the results are used to determine the analytical performances of the developed aptasensor. To the best of our knowledge, no label-free impedimetric aptasensor for the direct detection of small molecules such as CAP, reaching the MRPL value has been reported.

## 2. Experimental Section

### 2.1. Chemicals

CAP (MW = 323.13, CAS no. = 56-75-7) was purchased from Sigma (Diegem, Belgium). Tris buffer containing 100 mmol·L^−1^ sodium chloride, 20 mmol L^−1^ tris(hydroxymethyl)aminomethane, 5 mmol·L^−1^ potassium chloride, 2 mmol L^−1^ magnesium chloride and 1 mmol L^−1^ calcium chloride (pH = 7.6) was obtained from Sigma-Aldrich (Diegem, Belgium). CAP is practically insoluble in pure water. For this reason, the stock solution of CAP is prepared by means of ethanol and water (1:10). Potassium ferricyanide and ferrocyanide were purchased from Sigma. The CAP binding aptamer sequence (5′-SH-(CH_2_)_6_-AGC-AGC-ACA-GAG-GTC-AGA-TGA-CTG-AGG-GCA-CGG-ACA-GGA-GGG-GGA-GAG-ATG-GCG-TGA-GGTCCT-ATG-CGT-GCT-ACC-GTG-AA-3′) was purchased from Eurogentec (Seraing, Belgium) [11]. Before use, the aptamer solution was diluted in Tris buffer to a stock dilution of 100 µmol·L^−1^. The aptamer and CAP solutions were stored at 4 °C. All other chemicals and solvents were of analytical grade.

### 2.2. Apparatus

EIS measurements were performed by using a PGSTAT20 µAutolab Type III potentiostat controlled by Nova 1.9 software package equipped with FRA2 (ECO Chemie, Utrecht, The Netherlands). The electrochemical experiments were executed in a conventional three-electrode cell configuration. A gold electrode inlaid disk (Φ = 3 mm) was used as working electrode, a saturated calomel electrode (SCE) was used as the reference electrode and a platinum electrode was used as auxiliary electrode. All the potentials mentioned in the article are referred to the SCE. The electrochemical cell was placed in a Faraday cage to reduce electrical noise. All the EIS measurements (unless mentioned otherwise) were performed in a Tris buffer solution containing 10 mM K_3_[Fe(CN)_6_]/K_4_[Fe(CN)_6_] and under a potential of 10 mV (RMS) and around open circuit potential (OCP) over the frequency range 1–104 kHz.

### 2.3. Fabrication of the Sensing Interface

Prior to the aptamer immobilization, the gold electrode was mechanically polished with 1.0 and 0.05 µm alumina slurry and washed ultrasonically with deionized water. Subsequently, the gold electrode was chemically polished in a piranha solution to ensure the removal of all organic compounds from the surface. In a last stage, the gold electrode was electrochemically polished based on the procedure developed by Xiao *et al.* [17]. Briefly, the electrodes were cycled in 0.5 M sodium hydroxide solution between −350 and −1350 mV, at a scan rate of 2000 mV·s^−1^. Next, the surface of the gold electrode was pre-treated at +2000 mV for 5s and −350 mV for 10s in a 0.5 M sulfuric acid solution followed by sweeping the potential between −350 mV and +1500 mV at a scan rate of 4000 and 100 mV·s^−1^. Then, the electrodes were dipped in a 0.5 M sulfuric acid/0.1 M potassium chloride solution and the potential was cycled between +200 mV and +1500 mV with a scan rate of 100 mV·s^−1^. After the final preparation step, the electrodes were thoroughly rinsed with distilled water and dried at room temperature.

The thiolated aptamers were immobilized by dropping 6.5 µL of a 5 µM aptamer solution onto the freshly smoothed gold surface and electrodes were kept at +4 °C. Different incubation times for aptamers were investigated. The optimal incubation time of 4h was chosen based on the circumstances under which the polarization resistance change by CAP binding to aptamer was most effective in EIS analyses. The final aptasensing surface was ready after rinsing with a buffer solution.

## 3. Results and Discussion

### 3.1. The Electrochemical Behavior of a CAP-Aptamer Modified Electrode

**Figure 1.**
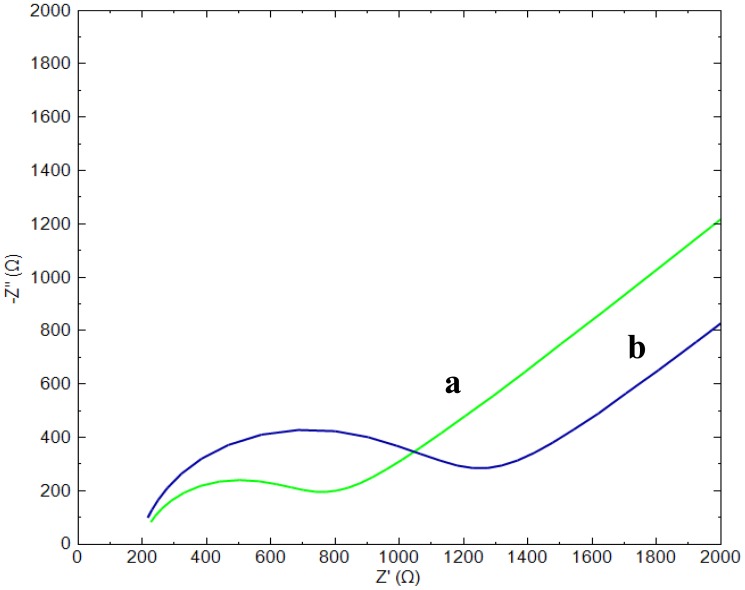
Nyquist plot of gold electrode before (**a**) and after aptamer immobilization (**b**) in the presence of 10 mM [Fe(CN)_6_]^4−/3−^ probe in Tris buffer solution of pH 7.6. The frequency was from 1 Hz to 1 kHz and the amplitude was 10.0 mV.

EIS measurements were employed to investigate the changes that occurred during the modification of the gold electrode. Figure 1 shows the Nyquist plots of [Fe(CN)_6_]^3−/4−^ for the bare gold electrode (a) and for a CAP-aptamer modified gold electrode (b).

The bare gold electrode shows a small semicircle domain in comparison to the modified gold electrode which is characteristic for a smaller charge transfer resistance (R_ct_), which is the polarization resistance at equilibrium potential, and hence a faster electron transfer [8]. The increased polarization resistance of the modified electrodes (curve b) is mainly due to the fact that the negative charges of the phosphate backbone of the aptamers create an electrostatic repulsive force to the negative redox couple [Fe(CN)_6_]^3−/4−^ anions. Due to this repulsion, the anions present in the structure of the probe experience a greater resistance to reach the surface which blocks the electron transfer of the redox probe. A second contribution to the increase of R_ct_ is the occupation of a part of the gold surface by the immobilized aptamers. In addition, un-modified electrodes show a straight line with a 41.9° angle at the low frequencies and Nyquist plot recorded for aptamer modified electrode shows a straight line with a slope of 41.6° angle. Deviation from ideal 45° slope depends on the homogeneity and roughness of surface [14].

### 3.2. EIS detection in the Presence of CAP

**Scheme 1.**
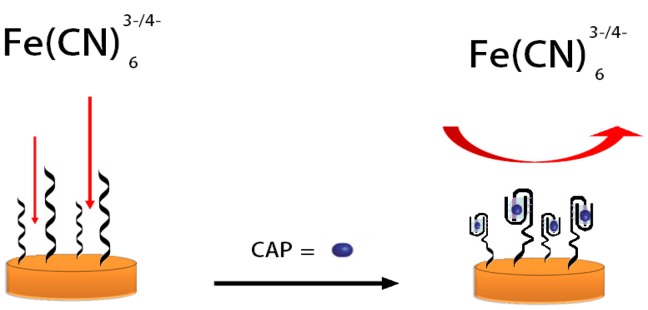
Electrochemical aptasensor based on label free target-induced folding of the aptamer.

Structural changes of oligunocleotides upon binding to the analyte affect the efficiency of the electron transfer to the electrode surface. In the absence of the CAP target molecule, the aptamer structure is partially unfolded. Upon binding of the target CAP, the aptamers will change their conformations structure which will change the double layer capacitance and charge transfer kinetics to the electrode surface. Binding of CAP to the aptamer undergoes target-induced folding and forms a stem-loop structure [17–19]. This adjustment has an effect on the polarization resistance and is detected by a changing interaction between the aptasensor and the redox active [Fe(CN)_6_]^3−/4−^ complex. Upon the conformational change, CAP molecules are forced close to the surface through an efficient interaction with the aptamers and block the surface from the redox couple which leads to an increase of the polarization resistance [19]. The detailed detection process is illustrated in Scheme 1.

The ratio between the polarization resistance upon target binding and the polarization resistance in the absence of the target was further used to describe a quantitative model according to Equation (1) [20]:

(1) $$\Delta R=\frac{R_{\text{probe}+\text{CAP}}}{R_{\text{probe}}}$$

**Figure 2.**
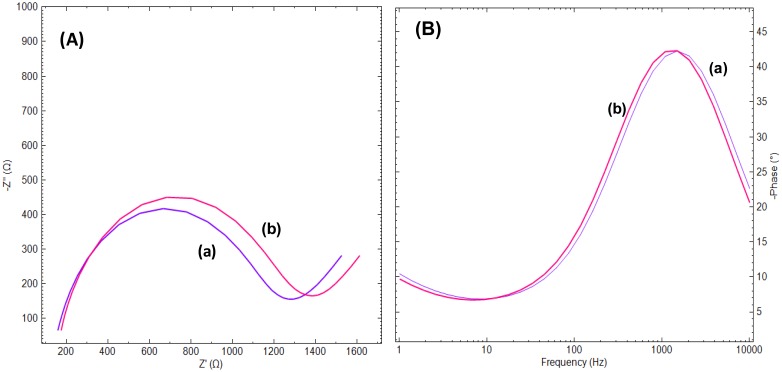
(**A**) Nyquist plots of the CAP-aptamer modified gold electrode in the absence (a) and in the presence of 90.6 nM CAP (b); (**B**) Bode plots of the CAP-aptamer modified gold electrode in the absence (a) and in the presence of 90.6 nM CAP (b) In the presence of 10 mM [Fe(CN)_6_]^4−/3−^ probe in Tris buffer solution of pH 7.6. The frequency range was 1 Hz to 1 kHz and the amplitude was 10.0 mV.

Figure 2A shows the Nyquist (A) and Bode (B) plots obtained at the CAP-aptamer modified gold electrode in the absence of CAP (curve a) and in the presence of 90.6 nM CAP (curve b). The interaction of the aptamer modified gold electrode surfaces with CAP molecules results in an increase of the phase as shown in the Bode plot (Figure 2B). This change is more pronounced at low frequencies while at high and intermediate frequencies, the two are overlapping. In the low frequency range the phase changes are dominated by the events in the molecular layer while at high frequencies the phase change is more sensitive to the composition of the buffer solution. As it was shown in the Nyquist plot, after interaction with the target solution, the conformational switch of the aptamer from linear to quadruplex and compaction of the surface layer structure results in decreasing the access of the electrode surface for ferricyanide ions added (curve b). However, the passivation of the gold electrode surface by means of alkanethiol molecules resulted in a noticeable decrease in R_ct_ of the aptamer modified electrode and presence of CAP molecules has no effect on R_ct_ value. This phenomenon could be due to the fact that presence of alkanethiols leads to the displacement of aptamers from the electrode surface [21] (data were not shown here).

### 3.3. Determination of the EEC for the Electrochemical Process

**Figure 3.**
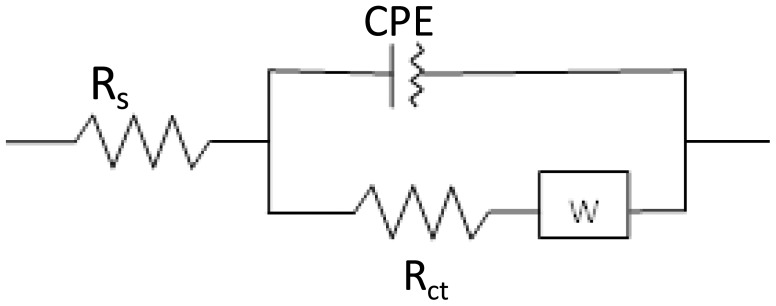
The EEC of the aptasensor.

As can be seen in Figures 1 and 2, the electrochemical process taking place during the sensing event is described by a slightly deformed semicircle followed by a straight line. A perfect semicircle is described by the well-know Randles circuit and forms the base of the EEC constructed for the aptasensor (Figure 3). The slight deformation of the circle is due to the fact that in real systems the double layer is never perfect due to the fact the electrode surface and ion layer at the surface are not completely homogenous and therefore the adsorption phenomena are not the same over the whole surface. To characterize this non-ideal behavior the standard Randles circuit is adapted by replacing the ideal capacitor by a constant phase element (CPE). The presence of the diffusion limitations at low frequencies must also be present in the circuit and is represented by a Warburg element (W). The obtained semi-circle indicates that multiple processes with different time constants occur. The high frequency values results from processes in the electrolyte or electrode bulk (conductivity), whereas the low frequency values result from processes at the electrode-electrolyte interface (double layer, charge transfer) or at the electrode surface (mass transport, adsorption, electrochemical reactions). Therefore, the specific interaction between probe and analyte has an effect on the impedance values at the lower frequency range. Since low enough values were chosen for the frequency range in the present study, it may result in a more sensitive detection of the analyte.

The EEC that describes the sensing event of the aptasensor is illustrated in Figure 3. The average chi-squared factor for this model was 0.0020 and is well below the limit for a good fitting between model and reality [22]. Hence, the chosen circuit was used to determine the value of the polarization resistance. The modelling and fitting was performed with the data-analysis Fit and Simulation of the Nova 1.9 software package running.

The model can also be used to determine the values of the parameters of CPE and W (Table 1). These parameters can be used to investigate and determine the double layer behavior and diffusion bound parameters such as the diffusion coefficient. The model does allow a differentiation between the several sub processes in one single measurement which is not possible with voltammetric measurements [16,20,23]. Chi-square test was performed and chi square factor (χ^2^) was used in order to evaluate the fitting between experimental and model data. The obtained values for χ^2^ are much lower than the tabulated value for 50 degrees of freedom (67.505 at the 95% confidence level), thus demonstrating the high significance of the final fits [22–25].

### 3.4. Analytical Performances of the Aptasensor for CAP

#### 3.4.1. Aptasensor Sensitivity and Linear Dynamic Range

**Figure 4.**
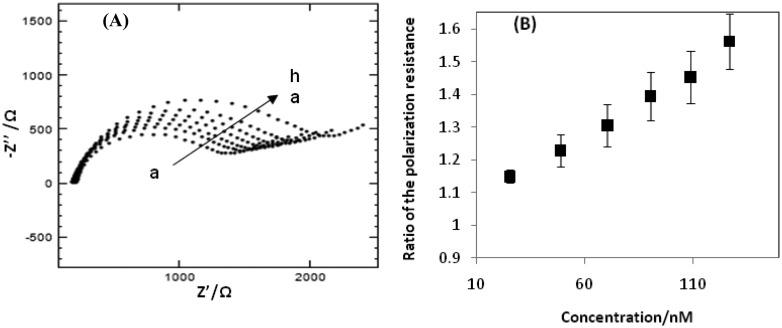
(**A**) Nyquist plots of the aptasensor in the solution containing different concentrations of CAP: 1.76, 25.4, 48.9, 70.5, 90.6 and 127 nM (from a to h) in the presence of 10 mM [Fe(CN)_6_]^4−/3−^ probe in Tris buffer solution of pH 7.6. The frequency was from 1 Hz to 1 kHz and the amplitude was 10.0 Mv; (**B**) The calibration plots of the increase of the ratio of the polarization resistance (ΔR) with the CAP concentration for the aptasensors.

The Nyquist plots obtained at the impedimetric aptasensor for different CAP concentrations are presented in Figure 4A. The higher the CAP-concentration present in the cell solution, the higher the polarization resistance was. Due to a higher concentration of CAP, a larger amount of aptamers undergo the target-induced folding leading to further blocking of the surface from the redox couple. Due to larger blocking of the redox couple, the electron transfer is further reduced, which is translated in a higher polarization resistance. To determine the relationship between the EIS response and the CAP concentration in a range of 1.76–127 nM, the ratio described in Equation (1) is applied. Figure 4B shows a good linear relationship between the ratio and the CAP concentration. The resulting linear equation was ΔR = 0.0039 C + 1.0404 with a correlation coefficient of 0.995. The lowest actually measured CAP concentration was 1.76 nM which closely reaches the MRPL value.

**Figure 5.**
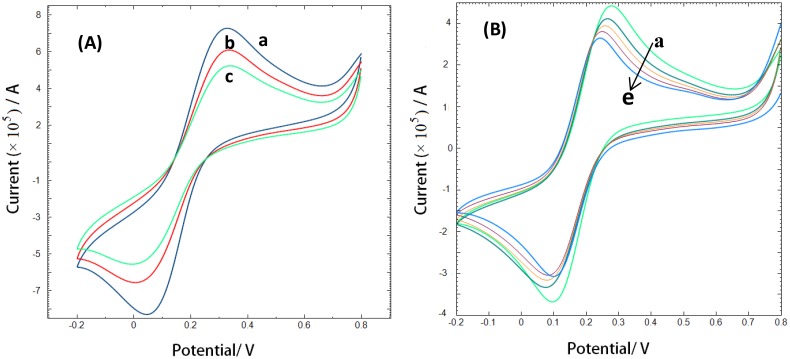
Cyclic voltammograms of 10 mM [Fe(CN)_6_]^4−/3−^ in 10 mM Tris buffer solution of pH 7.6 at scan rate of 50 mV/s for (**A**) bare gold electrode (a); CAP-aptamer modified gold electrode (b); CAP-aptamer modified gold electrode in the presence of 1.76 nM CAP (c); (**B**) CAP-aptamer modified gold electrode in the solution containing different concentrations of CAP: 1.76, 25.4, 48.9,70.5, 90.6 nM (from a to e).

Alternatively, cyclic voltammetric analysis was performed at CAP-aptamer modified electrodes which provide additional information about interface events. Figure 5 shows cyclic voltammograms of the oxidation/reduction of a 10 mM solution of [Fe(CN)_6_]^4−/3−^ couple in Tris buffer at bare gold electrode (a), CAP-aptamer modified electrode (b), and CAP-aptamer modified electrode in the presence of 1.76 nM CAP (c).

At the aptamer modified electrode, the electrochemical reaction of the redox probe is prevented and an increase in the peak-to-peak separation and a decrease in the peak current is observed. The self-assembly of the aptamer on the electrode surface forms a negatively charged interface that repels negatively charged [Fe(CN)_6_]^4−/3−^ anions. It is expected that the repulsion causes a slower interfacial electron-transfer kinetics of the redox probe. In the presence of target molecules, the bulky CAP molecules provide an additional barrier to the electron transfer of the redox species in solution and a further decrease of the peak current [12–14].

#### 3.4.2. Stability and Reproducibility

The stability of successive assays with the aptasensor was evaluated by performing five EIS measurements with a single electrode in Tris buffer with 10 mM redox couple after being incubated in 1.76 nM CAP (approximate measurement duration is 5 h). The result shows that the impidimetric response of CAP-aptamer modified electrode decreased only 19% after successive measurements. The high stability of the developed sensor can be ascribed to the fact that with EIS a much smaller perturbation of the system is applied, which reduces the effect of the measurement itself on the system [10,14,25]. In addition, the stability of the system suggests that the self-assembled aptamers form stable films that are not influenced by the measurement. This is an important advantage of the impedimetric sensor since the system can be perceived as stationary which can lead to simplifications upon interpreting the data. In addition, since an EIS measurement does not affect the system, the sustainability of the sensor is good.

The reproducibility of the aptasensor was investigated at the CAP concentration of 1.76 × 10^−9^, 25.4 × 10^−9^, 48.9 × 10^−9^, 70.5 × 10^−9^, 90.6 × 10^−8^, 100.9 × 10^−9^, 127 × 10^−9^ M and the obtained relative standard deviation for nine times of each concentration are 1.91%, 4.36%, 5.24%, 5.59%, 5.65%, 5.85%, 4.77%, respectively. The relative standard deviation (STD) for all measurements placed within the limit for reliable measurements (RSD < 5%) [26,27]. These results validate that the procedure is suitable for the development of a sensitive and reproducible aptasensor.

## 4. Conclusions

A novel, highly sensitive and stable label-free electrochemical aptasensor was developed for the detection of CAP and for the determination of the various subprocesses. The principle was grounded on the conformational change of the aptamers induced by binding of the CAP which leads to a distortion of the surface that is electrochemically detectable by EIS. Even though CAP is a small molecule, the present study demonstrates that EIS is a very stable and sensitive method for the detection of CAP. It has been shown that it is possible to investigate the electron transfer, the diffusion process and the double layer behavior within one single measurement. Furthermore, the developed sensor is effectively capable of detecting 1.76 nM CAP, approaching the MRPL. Unique properties of aptamers combined with the advantage of simplicity, sensitivity of impedimetric transduction, electrochemical impedimetric aptasensor may provide an alternative for the reliable detection of small molecules such as CAP over the classical analysis methods and with the advantage of the possibility for further fundamental studies based on the processes that take place.

## Figures and Tables

**Table 1.** Values of circuit elements obtained by fitting the experimental data from Figure 2A to the circuit model shown in Figure 3.

**Elements Type of Electrode**	**R_s_**	**R_ct_**	**W**	**CPE**	**χ^2^**
Unmodified gold electrode	193 Ω	550 Ω	197 μMho	712 nF	0.004
Aptamer modified electrode	200 Ω	900 kΩ	187 μMho	189 nF	0.05
